# Financial Development and Environmental Regulations: The Two Pillars of Green Transformation in China

**DOI:** 10.3390/ijerph17249242

**Published:** 2020-12-10

**Authors:** Cong Li, Xihua Liu, Xue Bai, Muhammad Umar

**Affiliations:** School of Economics, Qingdao University, Qingdao 266071, China; lic@qdu.edu.cn (C.L.); baixue1984@163.com (X.B.); umar@qdu.edu.cn (M.U.)

**Keywords:** China, environmental regulation, financial development, green technological progress, Shandong

## Abstract

Awareness of the influence of environmental regulations and financial development on green technological progress by Chinese enterprises will help to promote the green transformation of China’s economy, thereby comprehensively enhancing the quality and competitiveness of its economic development. This paper constructs a theoretical framework to analyze environmental regulation, financial development, and green technological progress and studies the relationship among these three indicators using 2004–2018 data from Shandong province. The results show that environmental regulations and financial development both play roles in promoting green technological progress, but as environmental regulation becomes stronger, the effects of finance on green technological progress begin to differ across regions. The results partially verify the applicability of the Porter hypothesis in China, providing a reference for all levels of government to formulate scientific and reasonable environmental rules and policies.

## 1. Introduction

Since the start of reform and opening-up, China has made remarkable achievements in economic development, with its economic aggregate ranking second in the world. However, the current development model, characterized by high levels of inputs, energy consumption, emissions, and pollution over a long period, make its economic growth excessively dependent on production inputs, seriously harming the environment and turning China into the world’s largest energy consumer and polluter (As is described in the Statistics of CO_2_ Emissions issued by the International Energy Agency (IEA), China’s total CO_2_ emissions reached 6.2 billion tons in 2007, causing China to surpass the US as the world’s largest carbon emitter; according to the BP Statistical Review issued by the British Petroleum Co. Ltd. (BP), China’s energy consumption accounted for 20.3% of the world’s total in 2010, surpassing the USA as world’s largest energy consumer). According to the “2018 Global Environmental Performance Index (EPI) Report”(The report was jointly published by the Center for Environmental Law and Policy at Yale University, Columbia University’s Center for International Geoscience Information (CIESIN) and the World Economic Forum (WEF)), China’s Environmental Performance Index in 2016 ranked the 120th among 180 countries and regions in the world with a score of 50.74, and ranked the 61st from the bottom among participating countries and regions. In order to resolve the problem of the environment, the Report of the 19th CPC National Congress clearly pointed out that “building an ecological civilization is a long-term project for the sustained development of the Chinese nation” and proposed that “the strictest ecological, environmental protection system be implemented.” In order to effectively balance the relationship between economic development and environmental protection, China is actively exploring new methods in green and low-carbon development in the economic and harmonious environment. The progress of green technology is the core tool for coordinating economic finance and the environment.

The innovation compensation effect is often used to indicate that appropriate environmental controls help companies innovate, thus improving their productivity and eventually offsetting the costs incurred by environmental protection, enhancing profitability [1,2,3].

The effectiveness depends on a company’s ability to make technological progress in production under environmental regulations, especially in high output, low energy consumption, and low emission production. Therefore, the key to achieving the dual goals of economic growth and environmental protection lies in the driving effect of environmental regulation on green technology development, but companies cannot make green technological progress without financial support [4,5,6]. With endowment advantages such as capital, markets, and credit, by directing the social capital flow, finance promotes innovation in and application of market-oriented tools, thereby solving problems such as shortage of behavioral incentives, unfavorable policy environments, and loss of essential tool operation factors, guiding social capital, establishing liquidity, and improving the credibility of firms to boost green technological progress and eventually achieve social welfare [7,8,9,10]. Therefore, financial supply is an important external factor for green technological progress.

However, further theoretical and empirical research is required on the combined action of environmental regulation and financial development on green technological progress and the internal mechanisms among the three. China has implemented a development strategy of factor-driven growth for quite some time, continuously increasing the marginal environmental costs of growth and weakening its drivers, which imposes significant pressure on environmental governance and economic transformation. Thus, further use of financial advantages to optimize environmental regulation to promote the combined positive action of both on green technology to achieve Potter’s effect has great significance for the green transformation of China’s economy, and the quality and competitiveness of economic development.

## 2. Literature Review

As environmental problems have become increasingly severe, discussion on the nexus between energy consumption, environmental challenges, and economic growth has taken center stage among stakeholders, policymakers, environmentalists, and government [11,12]. Some scholars study environmental challenges. They argue that innovation leads to technical change and has a positive impact on both economic growth and the environment through energy efficiency [13,14]. However, the literature cites some contradictory shreds of evidence as well. For example, Ahmed and Ozturk [15] argue that the establishment of smart grids and solar energy parks followed by energy sector reforms is yet to achieve plausible efficiency in China [14].

Some scholars have researched the detailed relationship between environmental regulations and green production technology, but their conclusions remain controversial. Grossman and Krueger [16] were the first to discover a U-shaped relationship between pollution and per-capita income, known as the “environmental Kuznets curve” hypothesis, which was later extensively verified [17,18,19]. Weimin and Lu [20] investigated the influence mechanism of China’s environmental regulations on green technological progress under the framework of directed technological change proposed by Acemoglu et al. [17], concluding that reasonable environmental regulation can change the direction of technological progress. Yongmei and Minjie [21] argued that environmental regulation has a U-shaped effect on green total factor productivity (GTFP): environmental regulation does not improve GTFP unless the ratio of pollution control costs to industrial value added is 3.8–5.1%. Tong et al. [22] argued that the influence of environmental regulation on the transformation and upgrading of industries forms a J-shaped curve, whose “knee” depends on the relative magnitude of resource allocation distortions and the technological effect of environmental regulation between pollution-intensive and clean industries. Yuan and Xiang [23] used the SYS-GMM method to show that environmental regulation positively impacts technological innovation in resource-intensive industries, negatively in labor-intensive industries, and has little effect in capital-intensive industries. Fan [24] found that a combination of incremental green taxes with government compensation effectively realized sustainable economic growth, and improved environmental quality and income distribution structure. In addition, some scholars have conducted research on the relationship between environmental regulations and green total production factors, and the research conclusions are still controversial. Lin and Xu [25] believe that the increase in the intensity of environmental regulations will also impose a mandatory “fine cleaning” on the industrial groups in the region, which will affect GTFP. Huang et al. [26] found that the impact of government environmental regulation policies on GTFP is time-sensitive and can effectively promote GTFP growth in the short term, but the long-term effect is just the opposite. Wang et al. [27] found an inverted U-shaped curve relationship between environmental regulations and green factor productivity based on panel data analysis of industrial sectors in OECD countries.

By mobilizing savings, increasing the return on invested capital (ROIC), improving liquidity, and spreading risks, financial development influences the efficiency of resource distribution and the development of technological innovations, promoting GTFP and long-term economic growth [28,29,30]. However, most existing research focuses on financial development and total factor productivity (TFP), and there are very few studies on financial development and green technological progress [31,32]. Rioja and Valev [33] found a nonlinear relationship between financial development and TFP with significant heterogeneity. Some scholars have argued that the misallocation of financial resources may reduce TFP, which indirectly supports the importance of financial development [34]. Weisheng [35] found that capital misallocation causes a loss of 18–33% in China’s manufacturing efficiency. Increasingly severe environmental problems have gradually turned the concept of green development into a focus of concern. Some scholars have begun to study the influence of financial development on GTFP [13,36,37,38,39]. Zhou et al. [39] argue that financial development can promote the growth of TFP and GTFP, but this effect diminishes as the level of financial development increases. Wang et al. [27] hold that financial development can promote the growth of GTFP through green technological progress, but this effect only appeared after 2008. Although these studies do not directly touch on green production technology, according to Fare [40], GTFP can be decomposed into green technological progress and green technological efficiency, which provides a high reference value for green technological progress research. Guan et al. [41] introduce technical factors under the basic framework of the Green Solow Model and analyze the changes in green total factor productivity under the background of environmental regulations. The research results show that green technological progress is the key to the improvement of green total factor productivity among regions, and it is reasonable. Environmental policies can stimulate the green technological progress of enterprises.

Few scholars have studied the relationship between environmental regulation, financial development, and the rate of green technology progress, and the research conclusions are still controversial. Wang and Sun [13] used panel data from 107 cities in the Yangtze River Economic Belt as a sample from 2003 to 2015. They found that financial development and environmental regulations have significantly promoted green technology progress and that financial development and environmental regulations impact green total factor productivity. The growth has a synergistic effect. A different conclusion comes from Ni et al. [42], who believe, based on the analysis of 30 provinces, municipalities, and autonomous regions in mainland China, that the combination of “environmental rules and financial development” has inhibited China’s green technological progress.

Thus, it can be seen that most existing studies focus on the relationship between environmental regulation and green technology progress and the relationship between financial development and green technology progress. However, the research conclusions are quite controversial due to the differences in research objects and research periods and the lack of incorporating regional financial development factors to consider the impact of environmental regulations on green technology progress. Simultaneously, more of the literature focuses on pure empirical analysis but less of the literature on theoretical analysis; more empirical analysis is based on provincial samples and samples from cities are rare.

In the economic and social field, the relationship between Shandong province and China is quite similar to China to the world. Shandong is positioned in China as a populous province with a shortage of resources, but at the same time, it has a dual identity of an economically developed industrial and agricultural province. Therefore, it is severely affected by resources and ecology in the process of green economic development, relying on the development of traditional heavy industry, and is subject to various restrictions in the restructuring and upgrading of its industrial structure, which is very similar to China’s position in the world. In terms of industrial structure, just like the huge differences between industries and regions that generally exist in China, there is also a huge imbalance between the eastern and western Shandong province regions. In addition, Shandong province has been actively implementing the central government’s various economic concepts, environmental protection measures, and green concepts in recent years. As a major economic, agricultural, and populous province in China, it has been practicing the concepts of green and environmental protection in recent years.

Considering the availability of sample data at the prefecture level, this paper studies the influence of environmental rules on green technology in terms of environmental regulation level, financial development, and green technological progress level using 2004–2018 data from Shandong Province, China. Financial development is introduced to build an “environmental rules–financial development–green development” mathematical model to study how environmental rules influence companies to progress in green technology. This paper explores whether environmental rules stimulate green technological innovation by promoting financial development, verifying the applicability of the Porter hypothesis (The “Porter Hypothesis” believes that appropriate environmental regulations can encourage enterprises to carry out more innovative activities, and these innovations will increase the productivity of enterprises, thereby offsetting the costs caused by environmental protection and improving the profitability of enterprises in the market product quality) in China. The paper makes the following possible research contributions. First, it puts environmental regulation, financial development, and green technological progress under a uniform research framework to analyze the interacting effects of environmental regulation and financial development on green technological progress; second, the model takes into consideration the ongoing supply-side structural reform with Chinese characteristics in order to evaluate its effect.

## 3. Theoretical Analysis

### 3.1. The Influence Mechanisms of Environmental Regulation

With strong negative externalities, environmental pollution not only causes market failure but also a great loss of social welfare. Therefore, in order to reduce the impact of environmental pollution and achieve social governance, the government usually sets a market access threshold by administrative means and sets environmental governance standards so that companies increase their environmental input. As a result, the unit output costs more. Environmental regulation can change an enterprise’s micro-internal behavior and macro-allocation efficiency through multiple channels, thereby indirectly influencing GTFP and promoting green technological progress. First, it can promote green production technology through technological innovations, so that companies proactively internalize the cost of environmental regulation to partly or fully offset the resulting increase in costs, to eventually achieve an innovation compensation effect. Second, it can promote market-based pricing to reflect the characteristics, supply, demand, and environmental cost of energy resources, preventing price distortion and upgrading the factor structure to improve TFP and finally increase the green technology level. Third, it promotes the transfer of essential productive factors from low-output, dirty industries to high-output, clean industries, upgrading the industry value chain to optimize scale benefits and comparative benefits brought about by industrial division, to improve GTFP and promote green technological progress.

### 3.2. The Influence Mechanisms of Financial Development

Financial development can influence green technological progress in the following ways. First, it can influence reasonable flow and optimum distribution of resources through the “invisible hand” of the market and the “visible hand” of the government to improve resource allocation efficiency and green technological productivity [13]. Second, it can provide essential financial support for technological innovation. Since it is, in essence, a channel for capital allocation across space and time, it can repurpose idle funds for technological innovation through credit to promote green technological progress. Third, it can provide investors with a means to spread risks and improve liquidity. Innovation is uncertain. A sound financial market can inter-temporally spread risks to improve companies’ technological innovation success rate. Sound financial mechanisms can provide companies with financing services to improve their liquidity, thus encouraging them to innovate.

### 3.3. The Synergistic Influence of Environmental Regulation and Financial Development

Environmental regulation and financial development are two important factors for green technological progress. First, with more environmental regulations, firms continuously increase their pollution management expenditure and technological R&D inputs, which inevitably generates great demand for funding, necessitating decentralization of R&D risks. If this demand fails to be met, they may be unable to improve TFP. Therefore, the support effect of finance plays a key role in improving GTFP. Second, environmental regulations also exert an expected effect on the flow of credit, motivating financial institutions to show more support for emerging industries, particularly high-tech ones, and also pushing conventional industries to transform themselves.

**Figure 1:**
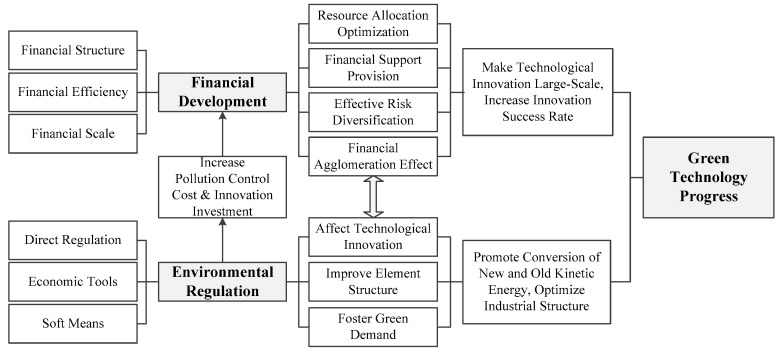
Influence mechanisms of environmental regulation and financial development on green technological progress.

The influence mechanisms of environmental regulation and financial development on green technological progress can be seen in Figure 1.

### 3.4. Theoretical Model For Influence of Environmental Regulation and Financial Development on Green Growth

In order to analyze the impact mechanism of environmental regulations on the progress of corporate green technology and the role of financial development in it, this paper builds a theoretical model that includes the financial supply of commercial banks based on the manufacturing progress function of enterprises. Since firms operate under certain environmental regulations, their pollution emissions cannot exceed the prescribed level. If pollution is generated during production, the pollution level is influenced by output. However, not all output produces pollution. See below:

(1) TW=ρTP

where TW represents the pollution amount, TP represents output, and ρ represents pollution emissions per unit production. In order to ensure that the pollution emission amount meets the regulatory requirements, part of the production costs include pollution control, so pollution management expenditure is also an important factor influencing the pollution level. A pollution function is created as shown below:

(2) Pol=W(TW,PC)

where Pol  represents the pollution level and PC represents the total pollution control expenditure.

Under the assumption of Hicks neutrality to technological progress, the firm’s pollution function is set as:

(3) $$TP=A(K_{A})f(K_{p})$$

where $A(K_{A})$ represents the production technology level, $f(K_{p})$ represents the output level at a given production technology level, $K_{A}$ represents technological capital input in production, and $K_{p}$ represents the other capital inputs in production besides technological capital. Since funding from the finance industry (especially commercial bank loans) is an important source of corporate capital inputs, given a financial supply level F:

(4) $$K_{A}=\phi \left(F_{A}\right),K_{p}=\varphi \left(F_{p}\right)$$

(5) ∂KA∂FA>0,∂Kp∂Fp>0

Under environmental regulation, the firm can control pollution in two ways. The first is direct, i.e., by increasing pollution control expenditure (PC). Suppose the firm manages pollution using funds from its total output. Pollution management expenditure α is expressed as follows:

(6) $$P\bar{C}=\alpha A(K_{A})f(K_{p})$$

where α represents the degree of the firm’s reaction to the environmental regulation intensity, 0<α<1.

The other way is indirect, i.e., through technological innovation. The firm can increase its output level. Although its emissions increase this way, its pollution management expenditure can be increased to reduce the impact of increased emissions, which is known as the output compensation effect. It can also transform and upgrade its product technology level and production mode through innovation to reduce pollution. This is known as the quality compensation effect. These two effects require innovations to compensate for environmental costs, so they are known as innovation compensation effects. The Porter hypothesis holds that the technological innovation effect of environmental regulation is primarily achieved through indirect channels. The two effects are assumed to be independent and separable from each other.

(7) $$A\left(K_{A}\right)=A_{1}\left(K_{A}\right)+A_{2}\left(K_{A}\right)$$

where $A_{1}\left(K_{A}\right)$ represents the output compensation effect and $A_{2}\left(K_{A}\right)$ represents the quality compensation effect. Owing to the action of $A_{2}\left(K_{A}\right)$, pollution emissions decrease rather than increase despite an in increase in output. Thus, pollution management expenditure decreases owing to the influence of $A_{2}\left(K_{A}\right)$. The following is a modified pollution management expenditure function:

(8) $$PC=\alpha \left(A_{1}\left(K_{A}\right)-\beta A_{2}\left(K_{A}\right)\right)f\left(K_{p}\right)$$

where β represents the amount of pollution management expenditure saved per unit $A_{2}\left(K_{A}\right)$, i.e., the marginal emission reduction effect of technology $A_{2}$. Accordingly, the emission amount is modified to be:

(9) $$TW=\rho \left(A_{1}\left(K_{A}\right)-\beta A_{2}\left(K_{A}\right)\right)f\left(K_{p}\right)$$

Moreover, by reference to the method proposed by Zhang et al. [6], it is assumed that the manufacturing progress function is $T_{A}\left(A_{1},A_{2}\right)$, so:

(10) $$T_{A}=T_{A_{1}}+T_{A_{2}}$$

Therefore, the profit function can be expressed as follows:

(11) $$\prod =P\left[\left(A_{1}\left(K_{A}\right)+A_{2}\left(K_{A}\right)\right)f\left(K_{p}\right)-\alpha \left(A_{1}\left(K_{A}\right)-\beta A_{2}\left(K_{A}\right)\right)f\left(K_{p}\right)\right]$$

The firm’s profit maximization behavior is:

(12) $$\{\begin{array}{c} Max\prod =P\left[\left(A_{1}\left(K_{A}\right)+A_{2}\left(K_{A}\right)\right)f\left(K_{p}\right)-\alpha \left(A_{1}\left(K_{A}\right)-\beta A_{2}\left(K_{A}\right)\right)f\left(K_{p}\right)\right]\\ \begin{array}{cc} s.t. & W\left[\rho \left(A_{1}\left(K_{A}\right)-\beta A_{2}\left(K_{A}\right)\right)f\left(K_{p}\right),\alpha \left(A_{1}\left(K_{A}\right)-\beta A_{2}\left(K_{A}\right)\right)f\left(K_{p}\right)\right]=R \end{array} \end{array}$$

The optimization conditions can be obtained from Formula (11):

(13) $$\{\begin{array}{c} P\left[\left(A_{1}{}^{\prime }+A_{2}{}^{\prime }\right)f\left(K_{p}\right)-\alpha \left(A_{1}{}^{\prime }-\beta A_{2}{}^{\prime }\right)f\left(K_{p}\right)\right]+\lambda \frac{\partial W}{\partial F_{A}}=0\\ -P\left(A_{1}-\beta A_{2}\right)f\left(K_{p}\right)+\lambda \frac{\partial W}{\partial \alpha }=0\\ W\left[\rho \left(A_{1}\left(K_{A}\right)-\beta A_{2}\left(K_{A}\right)\right)f\left(K_{p}\right),\alpha \left(A_{1}\left(K_{A}\right)-\beta A_{2}\left(K_{A}\right)\right)f\left(K_{p}\right)\right]=R \end{array}$$

The following can be derived from Formula (12):

(14) $$\frac{\partial W}{\partial PC}=-\rho \frac{\left(\frac{\partial A_{1}}{\partial K_{A}}\cdot \frac{\partial K_{A}}{\partial F_{A}}-\beta \frac{\partial A_{2}}{\partial K_{A}}\cdot \frac{\partial K_{A}}{\partial F_{A}}\right)}{\left(\frac{\partial A_{1}}{\partial K_{A}}\cdot \frac{\partial K_{A}}{\partial F_{A}}+\frac{\partial A_{2}}{\partial K_{A}}\cdot \frac{\partial K_{A}}{\partial F_{A}}\right)}\cdot \frac{\partial W}{\partial TW}$$

Suppose $M=\frac{\partial A_{1}}{\partial K_{A}}\cdot \frac{\partial K_{A}}{\partial F_{A}}$, $N=\frac{\partial A_{2}}{\partial K_{A}}\cdot \frac{\partial K_{A}}{\partial F_{A}}$. Substituting them into Formula (13):

(15) $$\frac{\partial W}{\partial PC}=-\rho \frac{\left(M-\beta N\right)}{\left(M+N\right)}\cdot \frac{\partial W}{\partial TW}$$

where $\frac{\partial W}{\partial PC}$ represents the marginal change in emissions from pollution control, $\frac{\partial W}{\partial TW}$ represents the marginal change in emissions from production, and $\frac{\partial A_{1}}{\partial K_{A}}\cdot \frac{\partial K_{A}}{\partial F_{A}}$ represents the marginal technological efficiency of funding supply. Thus:

(16) $$\frac{\partial W}{\partial A_{1}}=\rho f\left(K_{p}\right)\cdot \frac{\partial W}{\partial TW}+\alpha f\left(K_{p}\right)\cdot \frac{\partial W}{\partial PC}$$

(17) $$\frac{\partial W}{\partial A_{2}}=\left(-\rho \beta \right)f\left(K_{p}\right)\cdot \frac{\partial W}{\partial TW}+\left(-\alpha \beta \right)f\left(K_{p}\right)\cdot \frac{\partial W}{\partial PC}$$

Substituting Formula (14) into Formulas (15) and (16):

(18) $$\frac{\partial W}{\partial A_{1}}=\rho f\left(K_{p}\right)\cdot \left[1-\left(\frac{M-\beta N}{M+N}\right)\right]\cdot \frac{\partial W}{\partial TW}$$

(19) $$\frac{\partial W}{\partial A_{2}}=\rho \beta f\left(K_{p}\right)\cdot \left[\alpha \left(\frac{M-\beta N}{M+N}\right)-1\right]\cdot \frac{\partial W}{\partial TW}$$

where ρ>0, $f\left(K_{p}\right)>0$, $\frac{\partial W}{\partial TW}>0$, β>0, $\left[1-\left(\frac{M-\beta N}{M+N}\right)\right]>0$, and $\left[\alpha \left(\frac{M-\beta N}{M+N}\right)-1\right]<0$, so $\frac{\partial W}{\partial A_{1}}>0$, $\frac{\partial W}{\partial A_{2}}<0$. This shows that although better production technology ($A_{1}$) increases output, it can also aggravate pollution; progress in production technology ($A_{2}$) not only increases output but also reduces emissions.

Similarly:

(20) $$\frac{\partial W}{\partial F_{A}}=\rho \frac{\partial W}{\partial TW}\cdot f\left(K_{p}\right)\cdot \left(M-\beta N\right)\cdot \left[1-\frac{\alpha \left(M-\beta N\right)}{M+N}\right]$$

The above analysis indicates that the sign $\frac{\partial W}{\partial F_{A}}$ depends on (M−βN), that is, the marginal effect of the financial supply of technology and of the production technology on emissions reduction.

Because pollution emissions can reflect the intensity of environmental regulation, the production technology level function $T_{A}\left(A_{1},A_{2}\right)$ indicates that given a certain level of regulation:

(21) $$\frac{\partial T_{A}}{\partial A_{2}}=\frac{\partial T_{A_{2}}}{\partial W}\cdot \frac{\partial W}{\partial A_{2}}>0$$

Because $\frac{\partial W}{\partial A_{2}}<0$ and $\frac{\partial T_{A_{2}}}{\partial W}<0$, environmental regulation promotes progress in production technology $A_{2}$. In the inequality, $T_{A_{2}}$ reflects the level of green technological progress. Similarly, the following can be derived: $\frac{\partial T_{A_{1}}}{\partial W}>0$, $\frac{\partial T_{A_{2}}}{\partial F_{A}}>0$. This shows that stronger environmental regulation is not conducive to progress in production technology $A_{1}$, while more funding supply does promote green technology.

For further analysis, assume that:

(22) $$F_{A}=\theta F_{A_{2}}$$

where $F_{A_{1}}$ represents green production technology and financial supply and θ represents the marginal funding tendency. Thus,

(23) $$\frac{\partial T_{A_{2}}}{\partial F_{A_{2}}}=\frac{\partial T_{A_{2}}}{\partial F_{A}}\cdot \frac{\partial F_{A}}{\partial F_{A_{2}}}=\theta \cdot \frac{\partial T_{A_{2}}}{\partial F_{A}}$$

As can be seen, the effect of financial supply on green technological progress depends not only on the increase in total production amount but also on the marginal funding tendency.

In sum, stronger environmental regulation can promote advancement in green technology, more funding for green technological progress, and green technology level. The degree of the effect depends on the financial supply level and marginal funding tendency.

## 4. Model Selection and Specification of Variables

Based on the above results, this paper empirically analyzes the influence of China’s environmental regulation and financial development on its technological progress. The regression model is set up as follows:

(24) $$\begin{array}{l} \ln GTEC_{it}=\alpha_{0}+\alpha_{1}Fina_{it}+\alpha_{2}FEIR_{it}+\alpha_{3}Policy_{it}+\alpha_{4}Fina_{it}*FEIR_{it}\\ +\alpha_{5}Fina_{it}*Policy_{it}+\alpha_{6}FEIR_{it}*Policy_{it}+\alpha_{7}Wage_{it}+\alpha_{8}Edu+\alpha_{9}Tec_{it}\\ +\alpha_{10}Mar_{it}+\alpha_{11}Tra_{it}+\varepsilon_{it} \end{array}$$

where GTEC represents the green technological progress indicator, Fina represents the financial development level, FEIR represents the intensity of environmental regulations, Policy represents the supply-side reform policy, Fina∗FEIR represents the interaction of financial development and environmental regulation, Fina∗Policy represents the interaction of financial development and supply-side reforms, FEIR∗Policy represents the interaction of environmental regulation and supply-side reforms, Wage represents current employees’ average wage, Edu represents per capita education expenditure, Tec represents government support for technological innovation, Mar represents the market orientation level, and Tra represents the international trade level.

### 4.1. Intensity of Environmental Regulations

Weighted integration of multiple indicators (sewage, exhaust gas, and solid waste) is used to measure the intensity of environmental regulations [43]. The removal rate of sulfur dioxide, the removal rate of fumes and particulates (The removal rate of sulfur dioxide = (sulfur dioxide output-sulfur dioxide emissions)/sulfur dioxide output. The removal rate of fumes and particulates is calculated using the same method), and the comprehensive utilization rate of solid waste are processed using a weighted average to calculate environmental regulation intensity. The index is constructed as follows.

First, each indicator is given weight $w_{ij}\;$ and calculated as follows:

(25) $$w_{ij}=\left(\frac{E_{ij}}{\sum_{i=1}^{17}E_{ij}}\right)/\left(\frac{Y_{i}}{\sum_{i=1}^{17}Y_{i}}\right)=\left(\frac{E_{ij}}{Y_{i}}\right)/\left(\frac{\sum_{i=1}^{17}E_{ij}}{\sum_{i=1}^{17}Y_{i}}\right)$$

where *i* represents the administrative region, *j* represents the pollutant type, $E_{ij}$ represents the emissions of pollutant *j* in region *i*, and $Y_{i}\;$ represents the industrial added value of region *i*. Formula (25) can be understood as the ratio of emissions per unit industrial added value to the per-unit discharge amount of pollutant *j* in the region. The ratio can be used to measure the severity of emissions in a region.

After the weights $w_{ij}$ are constructed, the environmental regulation intensity can be calculated as follows:

(26) $$FEIR_{i}=\frac{1}{3}\sum_{j=1}^{3}w_{ij}PR_{ij}$$

where $PR_{ij}$ represents the removal rate of pollutant *j* in region *i* (also known as comprehensive utilization rate). Formula (26) indicates that environmental regulation intensity is positively correlated to the removal rate of pollutants, i.e., given a level of pollution emission intensity, the higher the removal rate, the higher the environmental regulation intensity; given a certain removal rate, the higher the emission intensity, and the higher the environmental regulation intensity.

### 4.2. Financial Development

In order to comprehensively consider financial development and prevent a single indicator from causing bias, with reference to the methodology of Wang et al. [13], this study uses financial scale, financial structure, and financial efficiency in its financial development system. Per capita deposits and loans are used to represent financial scale; the loan-to-deposit ratio is used to represent financial efficiency, and the ratio of total deposit and loan amount to GDP is used to represent financial structure (see Table 1 for details). An improved CRITIC evaluation method is used to calculate the weight of each indicator.

(27) $$W_{j}=\frac{\left(\sigma_{j}+e_{j}\right)\sum_{i=1}^{n}\left(1-r_{ij}\right)}{\sum_{j=1}^{m}\left(\sigma_{j}+e_{j}\right)\sum_{i=1}^{n}\left(1-r_{ij}\right)}$$

where $W_{j}$ represents the weight, $\sigma_{j}$ represents standard deviation, $r_{ij}$ represents the correlation coefficient, and $e_{j\;}$ represents information entropy.

### 4.3. Green Technological Progress

Green technological progress is decomposed from GTFP; therefore GTFP must be calculated first. The widely applied DEA-SBM is used here to calculate GTFP. The basic form of the SBM model is as follows:

(28) $$\begin{array}{c} \min\rho =\left[1-\frac{1}{m}\sum_{i=1}^{m}\left(S_{i}^{-}/x_{i0}\right)\right]/\left[1+\frac{1}{n}\sum_{\gamma =1}^{n}\left(S_{\gamma }^{+}/y_{\gamma 0}\right)\right]\\ s.t.x_{0}-X\lambda -S^{-}=0;y_{0}-Y\lambda +S^{+}=0;\lambda ,S^{-},S^{+}\geq 0 \end{array}$$

where S represents the input and output slacks, $S^{-}$ represents excessive input, $S^{+}$ represents insufficient output, λ represents the weighting vector, and 0 represents a given evaluation unit.

Taking undesirable output into consideration, the expression for a particular evaluation unit is changed to:

$$x_{0}=X\lambda +S^{-},y_{0}=Y^{\alpha }\lambda +S^{\alpha },Y_{0}^{\beta }=Y^{\beta }\lambda +S^{\beta }$$

Its planning form is:

(29) $$\begin{array}{c} \rho^{*}=\min\left\{\left[1-\frac{1}{m}\sum_{i=1}^{m}\left(S_{i}^{-}/x_{i0}\right)\right]/\left[1+\frac{1}{S_{1}+S_{2}}(\sum_{r=1}^{S_{1}}\frac{S_{\gamma }^{\alpha }}{y_{\gamma 0}^{\alpha }}+\sum_{r=1}^{S_{2}}\frac{S_{\gamma }^{\beta }}{y_{\gamma 0}^{\beta }}+)\right]\right\}\\ s.t.x_{0}=X\lambda +S^{+};y_{0}^{\alpha }=Y^{\alpha }\lambda -S^{\alpha };y_{0}^{\beta }=Y^{b}\lambda +S^{\beta };S^{-}\geq 0;S^{\alpha },S^{\beta },\lambda \geq 0 \end{array}$$

where $Y^{\alpha }$ represents desirable output, $y^{\beta }$ represents undesirable output, $S^{\alpha }$ represents insufficient desirable output, $S^{\beta }$ represents excessive undesirable output, and ρ represents the average ratio of actual input and output to inputs that could be reduced, and outputs that could be expanded with technology.

Based on the DEA-SBM model, as well as the construction method proposed by Chung et al. [44], the Malmquist GTFP productivity index is calculated using no economies of scale, as shown below:

(30) Mt+1(xt,yt,zt;xt+1,yt+1,zt+1)=[1+Dt(xt,yt,zt)1+Dt(xt+1,yt+1,zt+1)×1+Dt+1(xt,yt,zt)1+Dt+1(xt+1,yt+1,zt+1)]12=GTECt+1×ETECt+1=GTFPt+1

where $D^{t}\left(x_{t},y_{t},z_{t}\right)$ represents a directional distance function, GTFP represents green total factor productivity; GTEC represents the green technological progress index, and ETEC represents the green technological efficiency index. GTEC is the dependent variable, used to measure the level of green technological progress. The following is derived from Formula (26):

(31) GTECt+1=[1+Dt+1(xt,yt,zt)1+Dt(xt,yt,zt)×1+Dt+1(xt+1,yt+1,zt+1)1+Dt(xt+1,yt+1,zt+1)]12

Since GTFP must consider resources, energy, and environmental constraints, the inputs should include labor, capital, electric power, and water, while the outputs should also contain desirable outputs (total industrial output) and undesirable outputs (see Table 2).

### 4.4. Supply-Side Reform

Because it refers to a package of policies and measures, it is hard to find a single index to reflect supply-side structural reform. Considering the core status and important role of supply-side structural reform in overall economic and social reform, there must be a significant difference before and after the reform. Therefore, a dummy variable is set to substitute for supply-side reform, with reference to Zhang et al. [6], in order to analyze its effect on GTFP and its interactions with environmental regulations and financial development. Although supply-side reform was first proposed in 2015, it is aimed primarily at “eliminating unprofitable and polluting overcapacity, cutting excess inventory, deleveraging, reducing costs, and shoring up points of weakness.” Overcapacity is the most important item of all. Considering that the Shandong Provincial Government issued the Opinions on Implementing the Development Research Center of the State Council’s No. 41 Document (2013) for Resolving Overcapacity in the first quarter of 2014, the variable is set to 1 after 2014 and 0 before it. Thus:

(32) $$Policy_{it}=\{\begin{array}{c} \begin{array}{cc} 0 & t<2014\\ 1 & t\geq 2014 \end{array} \end{array}$$

### 4.5. Other Control Variables

(1) Human capital. Improvement in human capital may gradually strengthen a region’s capability to provide human resources for green technological progress. The average wage of current employees is used to measure human capital.

(2) Per capita education expenditure. Education is an important condition for the production and reproduction of science and technology, and educational advancement is inseparable from fiscal support. The ratio of government expenditure on education to registered population size is used in this paper to measure the degree of government support for education.

(3) Government support for technological innovation. Innovation can directly promote R&D in green production technologies, but it also needs fiscal support. Total fiscal expenditure for science and technology is used here to measure government support for technological innovation.

(4) Market orientation. Market orientation is critical to efficient resource allocation. It helps attract resources to R&D on green technologies, thus promoting green technological progress. The ratio of non-publicly owned to total industrial added value is used here to measure market orientation.

(5) International trade. The existence of comparative advantages is a contributor to the emergence of international trade, which helps different regions complement each other’s advantages and promotes the spread and progress of science and technology. The gross value of imports and exports is used here to measure international trade.

## 5. Data Selection and Empirical Analysis

### 5.1. Data Selection

The data used in this paper come from the Statistical Yearbooks of Shandong Province (2004–2018) and China Municipal Statistical Yearbooks (2004–2018). The data on municipal industrial sulfur dioxide emissions, industrial fume (particulate) emissions, industrial effluent volume, total industrial water supply, and total industrial power supply from the China Municipal Statistical Yearbooks (2004–2018), while all other data come from the Statistical Yearbooks of Shandong Province (2004–2018). All economic aggregate indicators are based on the household consumption level of Shandong Province of 2003, with the effect of price changes excluded. In order to eliminate the effect of factors like heteroskedasticity, variables including the average wage of current employees, per capita education expenditure, technological innovation input, and international trade are processed logarithmically.

### 5.2. Descriptive Statistics of Main Variables

Based on the above variable processing and index construction, descriptive statistics of the main variables are shown in Table 3.

### 5.3. Analysis of Regression Results

The general method of moments (GMM) [45] is used here to empirically analyze the panel data on Shandong province and its eastern and western halves (Shandong Province is divided into two parts according to per capita GDP at the end of 2016. Eastern Shandong consists of Jinan, Qingdao, Zibo, Dongying, Yantai, Weifang, Rizhao, Binzhou, and Weihai; western Shandong consists of Zaozhuang, Jining, Tai’an, Laiwu, Linyi, Dezhou, Liaocheng, and Heze. Although the categories are not geographical, the result is basically the same). This method remedies defects in conventional GMM, such as weak instrumental variables and sample information loss, so it can significantly improve the effectiveness and consistency of parameter estimation. It should be noted that this method is only effective in the absence of second-order autocorrelation among the residual terms and with valid instrumental variables. Therefore, the Sargan and Arellano–Bond tests are used to test the validity of the instrumental variables and the presence of second-order autocorrelation among the residual series (see Table 4).

The analyses in Table 4 of all of Shandong and both of its parts show that financial development and environmental regulation both promote green technological progress, i.e., it can be promoted by strengthening environmental regulation and increasing financial funding.

As indicated by the interaction effects of environmental regulation and financial development, the effect of financial support on green technological progress as environmental regulation becomes more stringent varies from region to region. It offers stronger support in eastern Shandong and weaker support in the west and the province as a whole. This result is primarily due to differences in financial development level, industrial structure, and technological innovation capacity between the different regions. As a traditional energy province, Shandong’s industrial structure tends to focus on traditional resource-based industries such as the light industry, chemical industry, and textiles. The secondary industrial structure is relatively large and the development of modern service industries is slow. These characteristics are particularly prominent in the western region. In recent years, the eastern region has developed a blue economy and an efficient ecological economy as the lead and has vigorously implemented the blue-yellow strategy. The region has a strip layout of cities, gives full play to the siphon effect, and provides sufficient popularity, logistics, and capital flows for the development of the entire region, and it also plays a key role in growing the economy. Therefore, eastern Shandong boasts a higher financial development level, a higher proportion of high-tech industries in the industrial structure, and stronger technological innovation capacity. Thus, firms are less affected by stronger environmental regulation, and their green technological R&D capacity remains strong. Additionally, green technological progress is increased due to the directed support for green firms under the guidance of green finance. However, western Shandong does not have such favorable conditions.

There are also regional differences in the influence of financial development and environmental regulation on green technological progress: there is much less influence in western than in eastern Shandong. The influence of supply-side reforms on the whole province and western Shandong is greater than in eastern Shandong. As the reforms deepen, financial development and environmental regulation support green technological progress more in western Shandong. However, since supply-side reform is a long-term task, its influence on green technological progress will remain significant in the short term in eastern Shandong.

### 5.4. Robustness Testing

The robustness of the estimation results is tested in the following ways. First, financial development is measured using financial concentration, with reference to the method proposed by Victor et al. (2011), and geographic density is used to measure financial concentration. The specific formula is as follows:

(33) $$LocalFin_{it}=\frac{Fin_{it}}{S_{it}}$$

where $LocalFin_{it}$ represents the financial development level of each city, $Fin_{it}$ represents its financial activity scale, and $S_{it}$ represents its geographical area. Data on year-end loan balances from all financial institutions in the district are used to measure the financial activity, and the administrative land area of the city (including the counties/districts and countryside) is selected.

Second, the input index is included in the total industrial gas supply of each city to estimate green technological progress, and desirable total industrial output is replaced with GDP to build a time-series panel model. The results show that although there are differences in significance level, the direction of most variables is consistent with the original model, verifying its robustness (see Table 5).

## 6. Conclusions

Reasonable environmental regulations can induce an “innovation compensation” effect, thereby covering enterprises’ compliance costs while improving their production capacity. Stronger regulation can help advance green technology, enhance financial support for green technological progress, and help increase the green technology level. The effect size depends on financial supply and the marginal financial support tendency. This paper has built a time-series panel data model to empirically test all of Shandong province and its eastern and western halves. The results show that (1) both financial development and environmental regulation positively influence green technological progress, i.e., green technological progress can be promoted by increasing financial supply and enhancing environmental regulation; (2) with more stringent environmental regulations, the influence of finance on green technological progress begins to differ by region. It provides stronger support in eastern Shandong, an economically developed region, and less support in the whole province and in western Shandong, which are economically undeveloped regions; (3) regional differences also exist in the influence of financial development and environmental regulation on green technological progress: they have much less influence in western Shandong than in the eastern half. The influence of the supply-side reform on the whole province and in western Shandong is greater than in eastern Shandong. As the reform deepens, financial development supports green technological progress more strongly in western Shandong, while environmental regulation also further increases green technological progress. However, since supply-side reform is a long-term task, its influence on green technological progress will remain significant in the short term in eastern Shandong.

Based on the above conclusions, this paper makes the following policy recommendations. First, promote the reform of Chinese commercial banks’ credit policy, actively developing green finance to coordinate the development of financial supply and environmental regulations. Financial institutions dominated by commercial banks should actively innovate credit models, expand and innovate at the original business level, continuously innovate and launch green credit products, use securities market tools to help green and environmentally friendly companies raise funds, and provide financing convenience for corporate green projects, meeting corporate financing needs. Second, improve China’s current environmental protection policies, implement differentiated environmental protection policies, and avoid “one size fits all” policies. On the basis of ensuring that the intensity of regulation does not drop, encourage the flexible combination of environmental regulation types, implement diversified incentive tools, and eliminate crude and straightforward “one size fits all” shutdown practices. Third, strengthen regional coordinated development and narrow the regional green technology gap. In view of the differences in the level of green technology in different regions, the government should focus on guiding exchanges and cooperation between regions, gradually narrowing the gap in regional development and achieving coordinated regional development. Economically developed regions should further increase investment in green technology and actively leverage technology spillover effects. Economic-backwardness regions ought to seize the historical opportunities and policy dividends of supply-side structural reformation and the conversion between old and new kinetic energy to promote green technology progress continuously.

## Figures and Tables

**Table 1:** Financial development index system.

Variables		Variable Calculation Method
Financial development	Financial scale	Total year-end deposits and loans/total registered population
	Financial efficiency	Year-end loan balance/deposit balance
	Financial structure	Total year-end deposits and loans/GDP

**Table 2:** Input and outputs.

Variables		Indicator
Inputs	Labor	Period-end number of employees per unit
	Capital	Annual average of net fixed assets
	Electricity	Industrial power consumption
	Water	Total industrial water supply
Desirable output	Economic output	Total industrial output value above designated size
Undesirable outputs	Pollution	Industrial effluent volume
		Industrial sulfur dioxide emissions
		Industrial fumes (particulate) emissions

**Table 3:** Descriptive statistics of main variables.

Variables	Observations	Mean	Standard Deviation	Minimum	Maximum
GTEC	238	0.9772	0.1132	0.6310	1.2850
Fina	238	0.3371	0.1198	0.1304	0.8752
FEIR	238	1.1366	0.9059	0.0588	5.5356
Fina*FEIR	238	0.3880	0.3678	0.0140	2.1914
Policy	238	0.2143	0.4111	0.0000	1.0000
Fina*Policy	238	0.0875	0.1805	0.0000	0.8752
FEIR*Policy	238	0.2930	0.7639	0.0000	4.9576
Wage	238	10.2547	0.5571	8.9591	11.2359
Edu	238	6.3314	0.8875	3.9723	8.0028
Tec	238	10.0586	1.1529	7.0175	12.5630
Mar	238	0.7212	0.1686	0.1774	0.9679
Tra	238	14.9640	1.2278	12.2180	17.7088

**Table 4:** Results of dynamic panel regression model estimation.

Variables	Whole Province: Model 1		Eastern Shandong: Model 2		Western Shandong: Model 3	
	Coefficient	Z-Value	Coefficient	Z-Value	Coefficient	Z-Value
L1.	0.098	1.51	0.054	0.54	−0.008	−0.08
L2.	−0.339 ***	−5.00	−0.380 ***	−4.22	−0.270 ***	−2.68
Fina	0.616 ***	2.81	0.681 ***	2.99	0.474 **	2.40
FEIR	0.218 ***	3.35	0.336 ***	5.35	0.132 *	1.69
Fina*FEIR	−0.528 ***	−3.26	0.436 ***	2.89	−0.342 *	−1.71
Policy	0.098 *	1.78	0.026	0.96	0.094 *	1.69
Fina*Policy	0.136 *	1.71	−0.705	−0.52	0.316 *	1.72
FEIR*Policy	−0.058	−0.92	0.032	0.88	0.221 *	1.79
Wage	0.274 ***	3.48	0.101	1.16	0.176	1.16
Edu	0.041*	1.93	0.092 **	2.28	−0.013	−0.37
Tec	−0.058	−1.64	−0.023	−0.52	−0.029	−0.52
Mar	−0.215 *	−1.74	0.131	1.39	−0.336 *	−1.67
Tra	0.003	0.11	−0.052	−1.34	0.077	1.07
Constant	−0.339	−0.67	−1.728 ***	2.92	−0.948	−1.22
Sargan Test	0.364		0.735		0.817	
AR (1)	0.053		0.042		0.036	
AR (2)	0.815		0.824		0.626	

Note: ***, **, and * mean significance at the 1%, 5% and 10% levels. The Sargan Test refers to the *p* value corresponding to the Sargan statistic measuring over-identification of the instrumental variables. AR(1) and AR(2) respectively refer to the P values corresponding to statistics from Arellano-Bond first-order and second-order serial correlation testing of the residuals after taking the first-order difference.

**Table 5:** Robustness test results.

Variables	Whole Province: Model 1		Eastern Shandong: Model 2		Western Shandong: Model 3	
	Coefficient	Z-Value	Coefficient	Z-Value	Coefficient	Z-Value
L1.	0.074	1.30	0.044	0.79	0.12	0.21
L2.	0.239 **	2.59	0.277 ***	3.27	−0.180 **	−2.65
Fina	0.416 *	1.81	0.563 ***	3.42	0.411 *	1.79
FEIR	0.318 ***	3.41	0.236 **	2.35	0.192 *	1.75
Fina*FEIR	−0.128 *	1.76	0.136 **	2.39	−0.042 *	−1.71
Policy	0.028 *	1.69	0.024	1.59	0.020	1.46
Fina*Policy	0.191 *	1.69	0.210	1.52	0.230 *	1.73
FEIR*Policy	0.078	1.32	−0.032	−0.78	0.132 *	−1.70
Wage	0.134	1.58	0.139 *	1.76	0.121	1.24
Edu	0.125 **	2.35	0.122 **	2.37	0.113	1.57
Tec	0.068	1.49	0.073 *	1.72	−0.019	−0.92
Mar	−0.310	−1.54	−0.231	−0.95	−0.311 *	−1.77
Tra	0.011	1.23	−0.032	−1.12	0.071 *	1.77
Constant	−1.401 *	−1.97	−1.828 *	−1.92	1.048	1.62
Sargan Test	0.462		0.533		0.711	
AR (1)	0.063		0.054		0.051	
AR (2)	0.235		0.311		0.199	

Note: ***, **, and * mean significance at the 1%, 5% and 10% levels.
